# A Functional Polymorphism in a Serotonin Transporter Gene (*5-HTTLPR*) Interacts with 9/11 to Predict Gun-Carrying Behavior

**DOI:** 10.1371/journal.pone.0070807

**Published:** 2013-08-28

**Authors:** J. C. Barnes, Kevin M. Beaver, Brian B. Boutwell

**Affiliations:** 1 School of Economic, Political and Policy Sciences, The University of Texas at Dallas, Richardson, Texas, United States of America; 2 College of Criminology and Criminal Justice, Florida State University, Tallahassee, Florida, United States of America; 3 Center for Social and Humanities Research, King Abdulaziz University, Jeddah, Saudi Arabia; 4 College of Criminal Justice, Sam Houston State University, Huntsville, Texas, United States of America; Radboud University, The Netherlands

## Abstract

On September 11, 2001, one of the deadliest terrorist attacks in US history took place on American soil and people around the world were impacted in myriad ways. Building on prior literature which suggests individuals are more likely to purchase a gun for self-protection if they are fearful of being victimized, the authors hypothesized that the terrorist attacks of 9/11 would lead to an increase in gun carrying among US residents. At the same time, a line of research has shown that a polymorphism in the *5-HTT* gene (i.e., *5-HTTLPR*) interacts with environmental stressors to predict a range of psychopathologies and behaviors. Thus, it was hypothesized that 9/11 and *5-HTTLPR* would interact to predict gun carrying. The results supported both hypotheses by revealing a positive association between 9/11 and gun carrying (*b* = .426, odds ratio = 1.531, standard error for *b* = .194, *z* = 2.196, *p* = .028) in the full sample of respondents (*n* = 15,052) and a statistically significant interaction between 9/11 and *5-HTTLPR* in the prediction of gun carrying (*b* = −1.519, odds ratio = .219, standard error for *b* = .703, *z* = −2.161, *p* = .031) in the genetic subsample of respondents (*n* = 2,350). This is one of the first studies to find an association between 9/11 and gun carrying and, more importantly, is the first study to report a gene-environment interaction (GxE) between a measured gene and a terrorist attack.

## Introduction

The terrorist attacks that occurred on September 11, 2001 (hereafter 9/11) sent shockwaves across the United States and throughout the world. Nearly every aspect of human life was touched by these events and, therefore, it is not surprising that scientists have uncovered large scale shifts in human behavior following 9/11 [1], [2]. Perhaps one of the most notable shifts to occur was the rise in levels of fear of terrorism among US residents. Gallup Poll results revealed, for instance, that only 24% of Americans were “very worried” or “somewhat worried” about being victimized by terrorism in April of 2000. This number jumped to 58% on the night of 9/11 and held steady around 50% for the next few months [2], [3]. Fear is a powerful human emotion originating in the evolutionarily ancient regions of the brain and corresponding to altered human behavior in a variety of ways [2]. Indeed, fear response varies from person to person with some responding aggressively and others responding passively. Because 9/11 was shown to raise fear levels for many Americans, scholars have begun to utilize 9/11 as a natural experiment to examine collective fear response patterns [2]. The result is that much is now known concerning the impact of 9/11 on collective behaviors such as governmental responses. What remains more elusive, however, is the degree to which 9/11 impacted individual-level fear-response behaviors (i.e., between-individual differences in behavior).

Even though fear of terrorism tended to increase, on average, across the majority of US citizens [3], the various ways in which people respond to fear in general, and to the fear of terrorism following 9/11 specifically, are highly variable. One of the more common ways to reduce fear of terrorism is to engage in self-protective behaviors, such as refusing to fly on an airplane, not traveling internationally, and maybe even purchasing a gun. This latter option is particularly unique because it is available to virtually all US citizens and it does not interfere with day-to-day living. Criminological research has revealed that a common reaction to the fear of victimization in general is to purchase and carry a handgun for self-protection [4]. Kleck and colleagues [4], for instance, reported that respondents who perceived more risk in their neighborhood were more likely to own a gun and were more likely to report intentions of purchasing a gun in the near future. Building on these results, we hypothesized that respondents would be more likely to report gun-carrying behaviors as a form of self-protection *after* 9/11 as compared to *before* 9/11 (hypothesis 1).

Though gun acquisition and gun carrying may be a viable form of self-protection, not all people respond to fear by purchasing and carrying firearms [4]. Thus, we should not expect all Americans to carry a weapon in response to 9/11 (or any other traumatic experience), but rather would expect individual-level traits and predispositions to partially moderate how people respond to fear of being victimized by a terrorist attack. A growing body of research has revealed one salient factor that has the potential to moderate how people respond to environmental stimuli is genotype. Gene-environment interactions (GxE), in general, run in one of two directions (other than a null result): a positive interaction or a negative interaction. A positive interaction—assuming a positive main effect of genotype on gun carrying—would suggest individuals with a certain genotype were more likely to carry a handgun after 9/11 as compared to before 9/11. A negative interaction—again assuming a positive main effect of genotype on gun-carrying—would suggest these individuals were more likely to carry a handgun before 9/11 and these differences were weakened, disappeared, or reversed after 9/11.

A polymorphism in the *5-HTT* gene has been shown to interact with environmental factors, including those that fall within the parameters of a fearful or traumatic experience, to produce different phenotypic outcomes [5]–[7]. A line of empirical research, for example, has suggested that *5-HTTLPR* interacts with stressful life events to predict depression [8](but see [9]), substance use [10], and decision making [11]. As such, we hypothesized *5-HTTLPR* would interact with 9/11 experience in predicting the self-protective behavior of gun carrying. It may be expected that individuals carrying the “risk” alleles for the *5-HTTLPR* polymorphism would be more likely to carry a handgun in response to 9/11 as compared to persons with other genotypes (hypothesis 2, a positive interaction). Note, however, that a negative interaction may also be expected; *5-HTTLPR* predicts handgun carrying prior to 9/11 but not after (hypothesis 3). The latter pattern of findings may emerge if 9/11 has an impact large enough to “overpower” any genetic predispositions (i.e., *all* people were more likely to carry a gun after 9/11, not just those with a genetic predisposition for this behavior).

## Methods

This research analyzes secondary data from the National Longitudinal Study of Adolescent Health (Add Health) [12]. Institutional Review Board approval to analyze the Add Health data was retained by all research team members from their respective institutions: The University of Texas at Dallas, Florida State University, and Sam Houston State University. The Add Health data have been described at length elsewhere [13], [14]. Briefly, the Add Health is a nationally representative longitudinal study of adolescents who were enrolled in middle or high school in 1995. Four waves of data have been collected with the most recent set of interviews being completed in 2008. Three key features of the Add Health study are important for the current analysis. First, wave 3 data collection took place between July 2001 and May 2002. Approximately 20% of all respondents were interviewed on or before 9/11/2001 (coded as 0) and all others were interviewed after 9/11/2001 (coded as 1) [15]. Sensitivity checks revealed that only eight total respondents (from the DNA subsample) were interviewed on 9/11/2001. The results of all analyses were identical to those presented here when these eight respondents were removed from the sample. None of these cases reported gun carrying.

The second feature of the Add Health data is that all respondents were asked the following question during wave 3 interviews: “In the past 12 months, how often did you carry a handgun at school or work?” Responses were dichotomized so that 0 = *never* and 1 = *at least once*. A total of 32 (1.35%) respondents (in the DNA subsample) reported carrying a gun to work/school. In order to avoid deductive disclosure due to fewer than 50 respondents reporting gun carrying, all case counts gleaned from cross-tabulations will be reported as percentages.

The third feature of the Add Health is that all twins and full siblings were genotyped during wave 3 interviews. Genotypic information was available for 2,574 respondents [16]. After eliminating one twin from each monozygotic twin pair (to avoid artificially decreasing standard errors) [17] and after eliminating cases with missing data, a final analytic sample of 2,350 was obtained. Respondents were genotyped for *5-HTTLPR*, which maps to 17q11.1-17q12. The *5-HTTLPR* polymorphism is the result of a 44 base pair (bp) VNTR in the 5′ regulatory region and two alleles have been identified: a short (S) allele (484 bp) and a long (L) allele (528 bp). The short allele has been associated with depression risk [7], lower life satisfaction [18], and with antisocial behavior [10]. Building on this literature, it was hypothesized that the short allele would be associated with self-protective behaviors and, therefore, *5-HTTLPR* was coded co-dominantly so that 0 = *no short alleles* (i.e., LL homozygotes; *n* = 797 [33.80%]), 1 = *one short allele* (i.e., SL heterozygotes; *n* = 1,082 [45.89%]), and 2 = *two short alleles* (i.e., SS homozygotes; *n* = 471 [19.97%]). Less than one percent of the analytic sample (0.34%) was missing genotypic information for *5-HTTLPR* and was coded as missing. Only one of these missing cases reported gun carrying. Substantive conclusions from the analysis were identical when *5-HTTLPR* was coded recessively (i.e., 0 = *no/one short allele*, 1 = *two short alleles*). When *5-HTTLPR* was coded dominantly (i.e., 0 = *no short allele*, 1 = *one or two short alleles*), the pattern and direction of findings were similar but conventional significance levels were not reached (*p* = .196 for the interaction term).

## Results

Preliminary analyses revealed an association between the gun carrying variable and 9/11 in the full sample of respondents (*n* = 15,052). A logistic regression model indicated respondents interviewed after 9/11 were 53% more likely to carry a gun to work/school as compared to respondents interviewed on or before 9/11 (*b* = .426, odds ratio = 1.531, standard error for *b* = .194, *z* = 2.196, *p* = .028). Results gleaned from the DNA subsample were substantively similar but the association did not reach statistical significance (*b* = .278, odds ratio = 1.321, standard error for *b* = .432, *z* = .644, *p* = .520).

Presented in Table 1 are results from a logistic regression model where the gun-carrying variable was utilized as the dependent variable and the 9/11 indicator variable, *5-HTTLPR*, and an interaction between 9/11 and *5-HTTLPR* (i.e., 9/11 * *5-HTTLPR*) were utilized as covariates (*n* = 2,350). Findings indicated 9/11 was associated with a marginally significant increase in gun carrying (*b* = 1.855, odds ratio = 6.392, standard error for *b* = 1.037, *z* = 1.788, *p* = .074), respondents with more short alleles on *5-HTTLPR* were more likely to carry a gun to work/school (*b* = 1.252, odds ratio = 3.499, standard error for *b* = .640, *z* = 1.958, *p* = .050), and the interaction between 9/11 and *5-HTTLPR* (i.e., 9/11 * *5-HTTLPR*) exhibited a negative and statistically significant impact on gun carrying (*b* = −1.519, odds ratio = .219, standard error for *b* = .703, *z* = −2.161, *p* = .031). The interaction term was negative, indicating that the influence of *5-HTTLPR* on gun carrying was diminished for respondents who were interviewed after 9/11.

**Table 1:** Logistic Regression of Gun Carrying on 9/11, *5-HTTPLR*, and an Interaction Term.

	Gun Carrying		
	*b*	*SE*	*p*
9/11 (0 = prior, 1 = after)	1.855	1.037	0.074
*5-HTTLPR* (0 = no short alleles, 1 = 1 short allele, 2 = 2 short alleles)	1.252	0.640	0.050
9/11 * *5-HTTLPR*	−1.519	0.703	0.031
*N*		2,350	

Note: Standard errors are adjusted to correct for the clustering of siblings within families.

**Figure 1:**
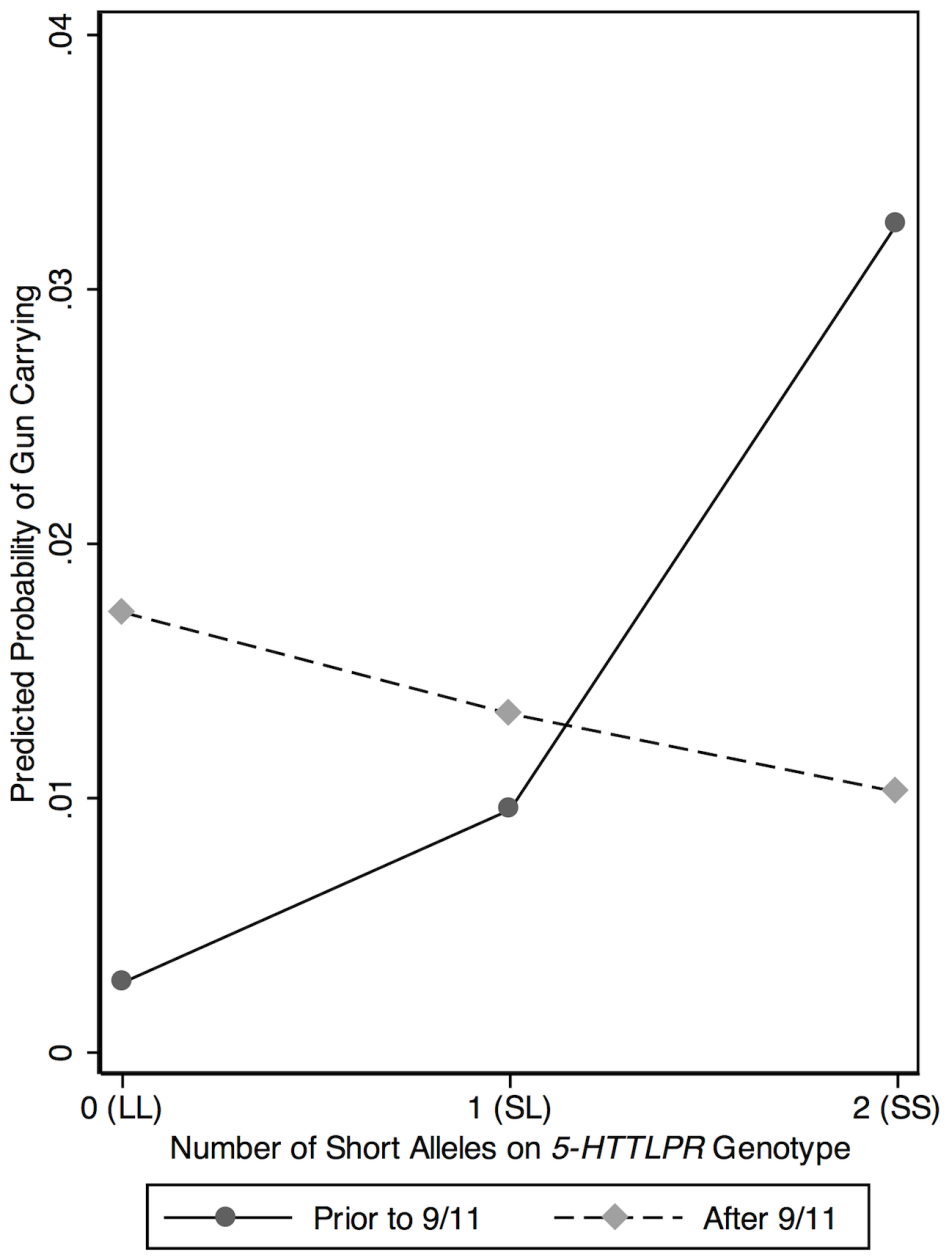
The Effect of *5-HTTLPR* on Gun Carrying Prior to and After 9/11.

The interaction between 9/11 and *5-HTTLPR* is plotted in Figure 1, which reveals that the predictive influence of *5-HTTLPR* is contingent upon whether the respondent was interviewed before or after 9/11. As shown in the figure, *5-HTTLPR* positively predicted gun carrying for respondents interviewed prior to 9/11/2001 (*n* = 632 total, 0.42% of LL homozygotes carried a gun, 0.71% of SL heterozygotes carried a gun, 3.57% of SS homozygotes carried a gun, *b* = 1.252, odds ratio = 3.499, standard error for *b* = .640, *z* = 1.957, *p* = .050). Respondents who were interviewed after 9/11/2001 showed no association between *5-HTTLPR* and gun carrying (*n* = 1,718 total, 1.61% of LL homozygotes carried a gun, 1.50% of SL heterozygotes carried a gun, 0.84% of SS homozygotes carried a gun, *b* = −.266, odds ratio = .766, standard error for *b* = .290, *z* = −.919, *p* = .358). A coefficient difference test [19] indicated that the effects of *5-HTTLPR* were significantly different across the two groups of respondents (*z* = 2.160, *p*<.05).

Three sensitivity analyses were conducted to test the robustness of the findings. First, the logistic regression model was re-estimated after controlling for the respondent's age, sex, and race. Given the association between racial/ethnic grouping and *5-HTTLPR* genotype [20], [21], the latter control seemed particularly important to include (self-reported racial categories were as follows: 72.05% of respondents self-identified as White, 18.71% self-identified as Black, 8.22% self-identified as Asian/Pacific Islander, and 5.39% self-identified as Native American). When these effects were included in the logistic regression model (race was included as a dummy variable identifying the respondent as Black), the main effect for the 9/11 variable was not statistically significant (*b* = 1.664, odds ratio = 5.278, standard error for *b* = 1.053, *z* = 1.580, *p* = .114), the main effect for *5-HTTLPR* was marginally significant (*b* = 1.208, odds ratio = 3.347, standard error for *b* = .650, *z* = 1.858, *p* = .063), and the 9/11 * *5-HTTLPR* interaction term was statistically significant (*b* = −1.523, odds ratio = .218, standard error for *b* = .706, *z* = −2.156, *p* = .031). Thus, the substantive conclusions of the interaction were largely unaffected by the inclusion of age, race, and sex controls.

The second sensitivity analysis included a control variable for the respondent's arrest history. Specifically, respondents were asked whether they had ever been arrested or taken into custody by police (coded 0 = *no* and 1 = *yes*). This variable was important to consider because an alternative explanation for the effect of the 9/11 variable may be that the most antisocial respondents (i.e., those who are likely to have been arrested in the past) are those who are also most likely to be interviewed later by researchers due to transience on part of the respondent. The arrest variable predicted gun carrying (*b* = 1.395, odds ratio = 4.034, standard error for *b* = .403, *z* = 3.457, *p* = .001) in a bivariate model suggesting some of the reported behavior may be illegal gun carrying. Also, the arrest variable predicted the 9/11 indicator in a bivariate model (*b* = .395, odds ratio = 1.485, standard error for *b* = .176, *z* = 2.248, *p* = .025), indicating the importance of including this variable as a statistical control. Importantly, the substantive conclusions drawn from the other variables were unchanged when the arrest variable was included. To be sure, the effect of the 9/11 variable did not reach statistical significance (*b* = 1.699, odds ratio = 5.467, standard error for *b* = 1.044, *z* = 1.627, *p* = .104), the coefficient for *5-HTTLPR* attained marginal significance (*b* = 1.192, odds ratio = 3.293, standard error for *b* = .638, *z* = 1.866, *p* = .062), and the 9/11 * *5-HTTLPR* interaction was statistically significant (*b* = −1.477, odds ratio = .228, standard error for *b* = .708, *z* = −2.086, *p* = .037). Virtually identical estimates were gleaned from a model that controlled for age, race, sex, and arrest history simultaneously.

The final set of sensitivity analyses re-estimated each of the logistic regression equations using the rare-events logistic regression model [22]. Because less than 2% of the sample reported gun carrying, it was important to re-analyze the associations outlined above with a statistical model that is able to account for low base rates on the dependent variable (i.e., limited 1 s as compared to 0 s). Overall, the substantive conclusions from the rare-events logistic regression models mirrored those outlined above. In terms of the 9/11 * *5-HTTLPR* interaction term, the rare-events model produced a negative and statically significant interaction in the base model (i.e., a model including the 9/11 variable, the *5-HTTLPR* variable, and the 9/11 * *5-HTTLPR* interaction) (*b* = −1.425, standard error for *b* = .702, *z* = −2.03, *p* = .042) and in a model with controls for age, race, sex, and arrest history (*b* = −1.434, standard error for *b* = .710, *z* = −2.02, *p* = .043). The split-sample models also were consistent with those reported above, but it is important to note the impact of *5-HTTLPR* was only marginally significant for the pre-9/11 cases in the rare-events logistic regression model (*n* = 632, *b* = 1.170, standard error for *b* = .638, *z* = 1.83, *p* = .067). Similar to the above, the impact of *5-HTTLPR* was not a statistically significant predictor of gun carrying for respondents interviewed post-9/11 (*n* = 1,718, *b* = −.254, standard error for *b* = .289, *z* = −.88, *p* = .379). A *z*-test [19] indicated the difference in coefficients was statistically significant (*z* = 2.03, *p*<.05).

## Discussion

Over the past decade, research has repeatedly shown genetic and environmental factors interact (GxE) in the prediction of human behavior [5], [23]. Working from this framework, we hypothesized that the events surrounding 9/11 would interact with a polymorphism in the *5-HTT* gene to predict gun-carrying behaviors (hypotheses 2 and 3). Results supported the *negative* interaction hypothesis (hypothesis 3) in two ways. First, a multiplicative interaction term between 9/11 and *5-HTTLPR* revealed a *negative* and statistically significant coefficient. Second, split sample models indicated *5-HTTLPR* was positively related to gun carrying before 9/11 but not after. Taken together, these results suggest a GxE between 9/11 and *5-HTTLPR* genotype and this pattern of findings indicates that certain persons (those carrying short alleles on *5-HTTLPR*) may have been more likely to carry a gun prior to 9/11 as a reflection of their greater likelihood to respond with self-protective behaviors to everyday situations. After 9/11, however, these differences were erased. These findings may be consistent with research indicating a greater impact of genetic factors on behavioral phenotypes in common or privileged environments as compared to disadvantaged or unpredictable environments [24], such as events that occur immediately following a terrorist attack.

The importance of these findings is twofold. First, to our knowledge, this is the first study to show an association between 9/11 and gun-carrying behaviors (hypothesis 1). Thus, the current results open a new line of inquiry into the myriad ways that 9/11 (and perhaps other terrorist attacks) affected US residents. Second, and perhaps more important, is that the current study is the first to test for a GxE with a measured polymorphism (*5-HTTLPR*) and a terrorist attack (9/11). GxE researchers have long noted the limitations of extant research, namely that environmental factors may be contaminated with genetic influences (via gene-environment correlations). In other words, a test of GxE may actually reflect a gene-gene interaction (i.e., GxG). By utilizing a terrorist attack as an exogenous environmental influence, we removed all possibility that the GxE (i.e., 9/11 * *5-HTTLPR*) was the result of a GxG. In short, this study presents what may be the purest way to test for GxEs. A related alternative would be to test for GxEs with natural disasters acting as the environmental influence [25].

Limitations to the analysis must be considered. First, though the sample size was large (*n*>2,000 for the DNA subsample), less than 40 respondents reported gun-carrying behaviors. This may have limited the statistical power of the analysis and most certainly inflated standard errors. Note that the conventional alpha level of .05 was utilized in order to limit the possibility of false-positives (Type I error) and that the rare-events logistic regression model produced a pattern of findings similar to those garnered from the standard logistic regression model. Nonetheless, some of the analytic cells had low case counts, meaning that the findings should be interpreted with due caution until replicated on an independent sample.

The second primary limitation concerns the wording of the gun carrying question. Specifically, respondents were asked to report on whether they had carried a gun over the past 12 months. This means respondents interviewed *after* 9/11 may have reported on gun carrying prior to 9/11. It will be important for future work to replicate these findings using alternative samples not subject to this limitation to determine whether, and to what extent, this may have impacted the results. Additionally, it is important that scholars consider whether alternative explanations exist for why gun carrying should differ across the two groups of respondents. The most likely argument seems to be that respondents interviewed later in the year or in 2002 may have been systematically different than those interviewed earlier in the wave 3 process due to location issues and the difficultly of tracking down transient and perhaps antisocial respondents. We explored this possibility by including a control variable for prior arrest history. The substantive findings for the interaction were unchanged when this variable was included suggesting this alternative explanation is not viable. For now, the results of our study suggest that 9/11 is an exogenous environmental pathogen that can be used in future GxE research to examine a range of phenotypic outcomes.
